# Genetic variation in *NDFIP1* modifies the metabolic patterns in immune cells of multiple sclerosis patients

**DOI:** 10.1038/s41598-021-00528-8

**Published:** 2021-11-01

**Authors:** Pilar López-Cotarelo, Adela González-Jiménez, Teresa Agudo-Jiménez, Judith Abarca-Zabalía, Yolanda Aladro, Belén Pilo, Manuel Comabella, Laura Espino-Paisán, Elena Urcelay

**Affiliations:** 1 Laboratorio de Investigación en Genética y Bases Moleculares de Enfermedades Complejas, Instituto de Investigación Sanitaria del Hospital Clínico San Carlos (IdISSC), Madrid, Spain; 2 Neurology Department, Hospital Universitario de Getafe, Madrid, Spain; 3 Servei de Neurologia-Neuroimmunologia, Centre d’Esclerosi Múltiple de Catalunya (Cemcat), Institut de Recerca Valld’Hebron (VHIR), Hospital Universitari Valld’Hebron, Universitat Autònoma, Barcelona, Spain; 4 Red Española de Esclerosis Múltiple (REEM), Madrid, Spain

**Keywords:** Genetic markers, Immunogenetics, Autoimmunity, Gene regulation in immune cells, Immunogenetics, Neurological disorders, Multiple sclerosis

## Abstract

One of the 233 polymorphisms associated with multiple sclerosis (MS) susceptibility lies within the *NDFIP1* gene, and it was previously identified as eQTL in healthy controls. NDFIP1 shows interesting immune functions and is involved in the development of the central nervous system. We aimed at studying the *NDFIP1* variant on activation and metabolism of immune cells. *NDFIP1* mRNA and protein expression were assessed in PBMCs by qPCR and western blot in 87 MS patients and 84 healthy controls genotyped for rs4912622. Immune activation after PHA stimulation was evaluated by CD69 upregulation, and metabolic function of both basal and PHA-activated lymphocytes was studied by Seahorse Xfp-Analyzer. In minor-allele homozygous controls but not in patients, we found higher *NDFIP1* expression, significantly reduced protein levels, and CD69 upregulation in B- and T-cells. PBMCs from minor-allele homozygous controls showed significantly higher basal mitochondrial respiration and ATP production compared to major-allele carriers, while minor-allele homozygous patients showed significantly lower metabolic activity than carriers of the major allele. In conclusion, we describe associations in minor-allele homozygous controls with lower levels of NDFIP1 protein, CD69 upregulation, and raised mitochondrial activity, which are not replicated in MS patients, suggesting a *NDFIP1* differential effect in health and disease.

## Introduction

Multiple Sclerosis (MS) is a chronic, demyelinating, inflammatory, immune-mediated disease with an increasing prevalence since 1955, and approximately 2.5 million patients worldwide^1^. The aetiology of the disease is still elusive, albeit the most accepted model proposes a combination of genetic and environmental factors. For decades, the only genetic risk factors for MS were located in the Major Histocompatibility Complex (MHC) locus. More recently, genome-wide association studies (GWAS) have identified over 230 MS risk polymorphisms,which account for 19.2% of the total heritability^2^. Despite the massive knowledge provided by GWAS and subsequent meta-analyses, the function of most of these variants associated with MS susceptibility remains unknown, and efforts are focused on explaining their relevance. A similar scenario exists for the environmental risk factors, with only vitamin D levels, obesity in adolescence, tobacco, and Epstein Barr virus with a proven association to MS, but with other prevalent viral infections (human Herpes virus or citomegalovirus, among them) in open debate^3^.

One of the MS-risk SNPs identified by GWAS is rs1036207^4^, located in the first intron of *NDFIP1*. Polymorphisms in the *NDFIP1* gene have been also associated with other inflammatory and autoimmune diseases^5^. A recent study in blood from healthy controls reported rs1036207 as an eQTL (expression Quantitative Trait Locus) of the gene, with the minor allele displaying a reduced *NDFIP1* expression^6^.

The Nedd4 Family Interacting Protein 1 (NDFIP1), a transmembrane protein located in Golgi and cytoplasmic vesicles, acts as an adaptor recruiting Nedd4 E3 ligases to specific substrates, and activating E3 ubiquitin ligases. The ubiquitin system is a key regulatory mechanism for cellular processes including protein turnover, signal transduction, and cell-cycle control. Approximately 5% of the human genome encodes more than 600 E3 ligases, and 13% of them are mutated in neurological disorders^7^.

NDFIP1 plays an important role in regulation of both immune and nervous systems. Numerous studies in animal models indicate that Ndfip1 is involved in several neuronal processes such as dendrite patterning, neurite outgrowth and cortical development^8^. Moreover, lower expression of *Ndfip1* has been associated with the pathogenesis of Alzheimer disease^9^, while it induces neuroprotection following neuronal injury or stress^10^. Additionally, through interaction with its downstream E3 ligases, NDFIP1 plays key roles in regulating peripheral tolerance^11,12^ and T-cell differentiation and maturation. In mice, CD4^+^ T cells lacking *Ndfip1* present deficiencies in their differentiation into T helper (Th) 2 or Th17 cells, as well as negligent cell cycle exit to arrest; while *Ndfip1*-deficient CD8^+^ T cells display a breach of tolerance to abundant common antigens^13^. Increased pro-inflammatory autoreactive T cells, i.e. Th17 and Th1 cells, and decreased number and impaired function of regulatory T cells characterize the immune profile of MS^14^. The metabolic signatures of T cells are closely related to their differentiation and activation status. Distinct metabolic programs are essential for the survival and specification of effector (Teff) and regulatory (Treg) T cells, as Teffs require a glycolytic metabolism whereas Treg depend on lipid oxidation. Upon activation, *naïve* T cells transform in proliferative effector T cells and switch from oxidative phosphorylation to aerobic glycolysis to meet the increased demand for cellular energy and biomass^15^. Maintenance of immune homeostasis requires exquisite control. The immune system needs to be both sufficiently aggressive to eradicate cells that express foreign antigens, and yet provide tolerance to self-antigens.

Our study aimed to examine the role of this genetic variation in *NDFIP1* in both healthy controls and MS patients. We compared the metabolic program of peripheral blood mononuclear cells obtained from MS patients and healthy controls to further our understanding of MS aetiology and to provide clues for the treatment of this autoimmune disorder associated with T cell dysfunction, as targeting cell metabolism may provide new avenues to suppress immunity.

## Results

**Figure 1:**
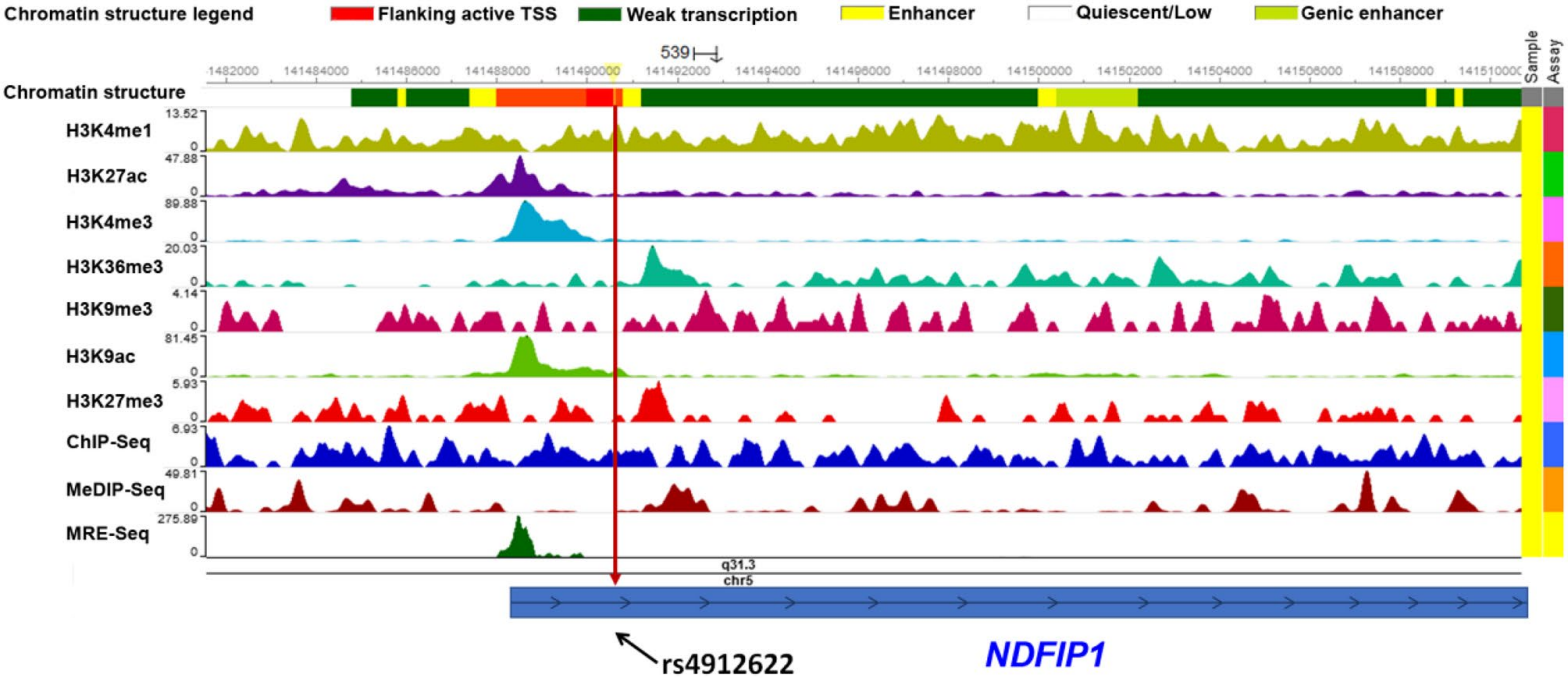
Roadmap epigenomics output for rs4912622. Chromatin structure and main epigenetic marks in the region surrounding rs4912622. Adapted from the Roadmap epigenomics project (http://www.roadmapepigenomics.org/).

The rs1036207 polymorphism has been identified as an MS-risk variant by GWAS and described as an eQTL for *NDFIP1* in whole blood from healthy controls^6^. Thus, we aimed to study the influence of this *NDFIP1* variant in MS patients compared to controls. Through “in silico” analyses, we observed a linkage disequilibrium block at the 5’ end of the *NDFIP1* gene which includes 65 SNPs with r^2^ ≥ 0.9 and D’ = 1 with rs1036207 (Table S1). Among them, one located in the first intron of the gene, rs4912622, reached the best regulatory score according to RegulomeDB data^16^. In PBMCs, and considering the information provided by the Roadmap Epigenomics Project^17^ (Fig. 1), the epigenomic profile of the region surrounding rs4912622 indicates high levels of histone marks such as H3 lysine 4 monomethylation (H3K4me1), H3 lysine 27 acetylation (H3K27ac) and H3K36me3, signs of chromatin accessibility, presence of enhancers, and active transcription, respectively. Regarding DNA methylation, the analyses of MEDIP-Seq and MRE-seq showed low levels of DNA methylation close to rs4912622. In summary, this MS-risk SNP lies in a region with evidence of regulatory activity, and therefore it was selected as a proxy for rs1036207 in the present study. In our samples, the minor allele rs4912622*G presented frequencies of 0.29 in healthy donors and 0.38 in MS patients, in line with those reported by The 1000 Genomes Project^18^.

**Figure 2:**
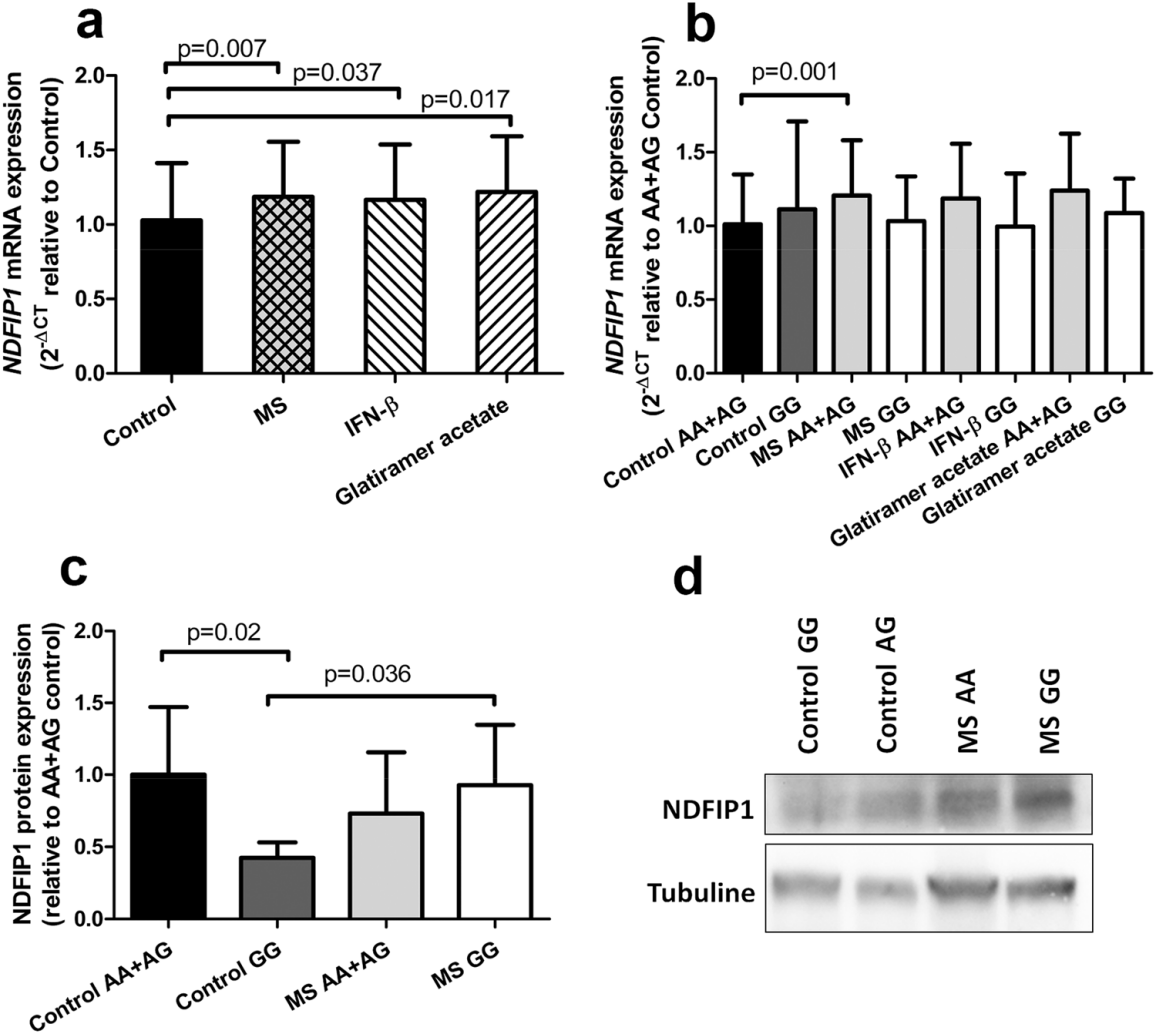
Differential effect of rs4912622 genotypes on *NDFIP1* mRNA and protein expression. (**A**) *NDFIP1* expression in healthy donors (n = 83) and MS patients (n = 87). (**B**) Allele specific effect of rs4912622 on *NDFIP1* expression (control AA + AG: n = 71; control GG: n = 13; MS AA + AG: n = 77; MS GG: n = 10). (**C**) Effect of rs4912622 on NDFIP1 protein level (control AA + AG: n = 12; control GG: n = 4; MS AA + AG: n = 16; MS GG: n = 4). Means ± standard deviation are shown. (**D**) Western blot of NDFIP1 with an individual of each genotype group included in (**C**). Full-length blots are presented in Supplementary Fig. 1.

We then analysed *NDFIP1* expression in PBMCs isolated from MS patients and healthy controls. As shown in Fig. 2A, significantly higher levels of *NDFIP1* mRNA were found in MS patients compared to healthy controls (*p* = 0.007). Concerning the allele-specific effect in mRNA expression (Fig. 2B), we observed opposite trends in the genotypes in controls and patients: while the minor-allele homozygotes showed a trend to have a lower *NDFIP1* expression in MS patients compared to controls, the major allele carriers had a higher expression in MS patients than in controls, a difference that was statistically significant (*p* = 0.001). In the control population, a trend to lower expression in major-allele carriers was observed when compared to GG homozygotes, although it did not reach statistical significance. Interestingly, the opposite trend was found in MS patients, and it was still present when interferon β or glatiramer acetate treated patients were analysed separately. No significant differences were evidenced between the two MS treatments used (β-Interferon or glatiramer acetate), neither in the overall MS population nor in the allele-stratified analyses, so we discarded a specific effect of a particular treatment in the described results.

To test whether the SNP was affecting NDFIP1 protein expression, the PBMCs content was analysed by Western blot. Remarkably, the MS-risk polymorphism exhibited an opposite effect to that observed in mRNA expression. Figure 2C,D show that the protective genotype induced a reduction to half the protein levels compared to major-allele carriers in healthy controls (*p* = 0.02), while no significant differences between genotypes were observed in MS patients.

**Figure 3:**
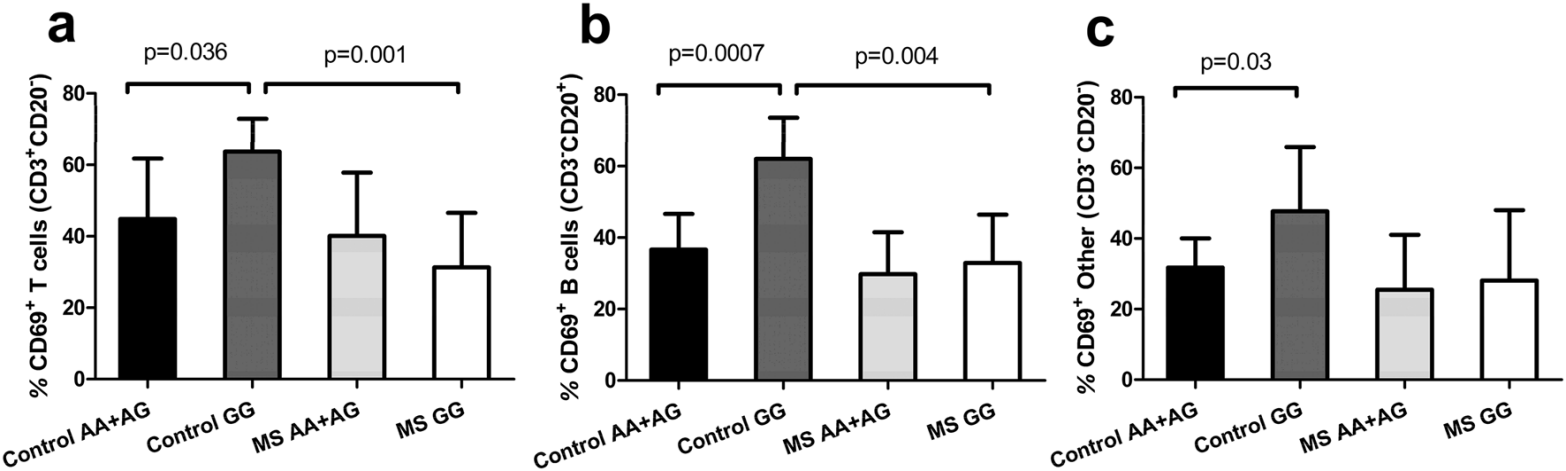
Lymphocyte activation in healthy controls and MS patients stratified by rs4912622 genotype. Effect of rs4912622 genotypes on surface expression of CD69 in (**A**) CD3^+^ CD20^-^ lymphocytes (control AA + AG: n = 11; control GG: n = 5; MS AA + AG: n = 16; MS GG: n = 8); (**B**) CD3^-^ CD20^+^ (control AA + AG: n = 10; control GG: n = 5; MS AA + AG: n = 15; MS GG: n = 6). (**C**) CD3^-^CD20^-^ (control AA + AG: n = 10; control GG: n = 5; MS AA + AG: n = 16; MS GG: n = 8).  Means of the percentage of cells expressing CD69 ± standard deviation are shown.

After stimulation of PBMCs with phytohemagglutinin (PHA), lymphocyte activation was assessed by flow cytometry analysis of CD69 upregulation (Fig. 3). Comparable results were observed both in T and B lymphocytes (Fig. 3A,B), which showed statistically significant higher activation status in minor-allele homozygous controls than in MS patients with the same genotype (*p* = 0.001 and *p* = 0.004 for T- and B-cells, respectively). In healthy controls, minor-allele homozygotes evidenced higher activation than that observed for carriers of the major allele (*p* = 0.036, *p* = 0.0007 and *p* = 0.03 in T-, B-cells and also in the CD3^-^CD20^-^ lymphocyte population, which is mostly composed of NK cells, respectively), while no significant differences among genotypes were found in MS patients (Figs. 3A-C).

**Figure 4:**
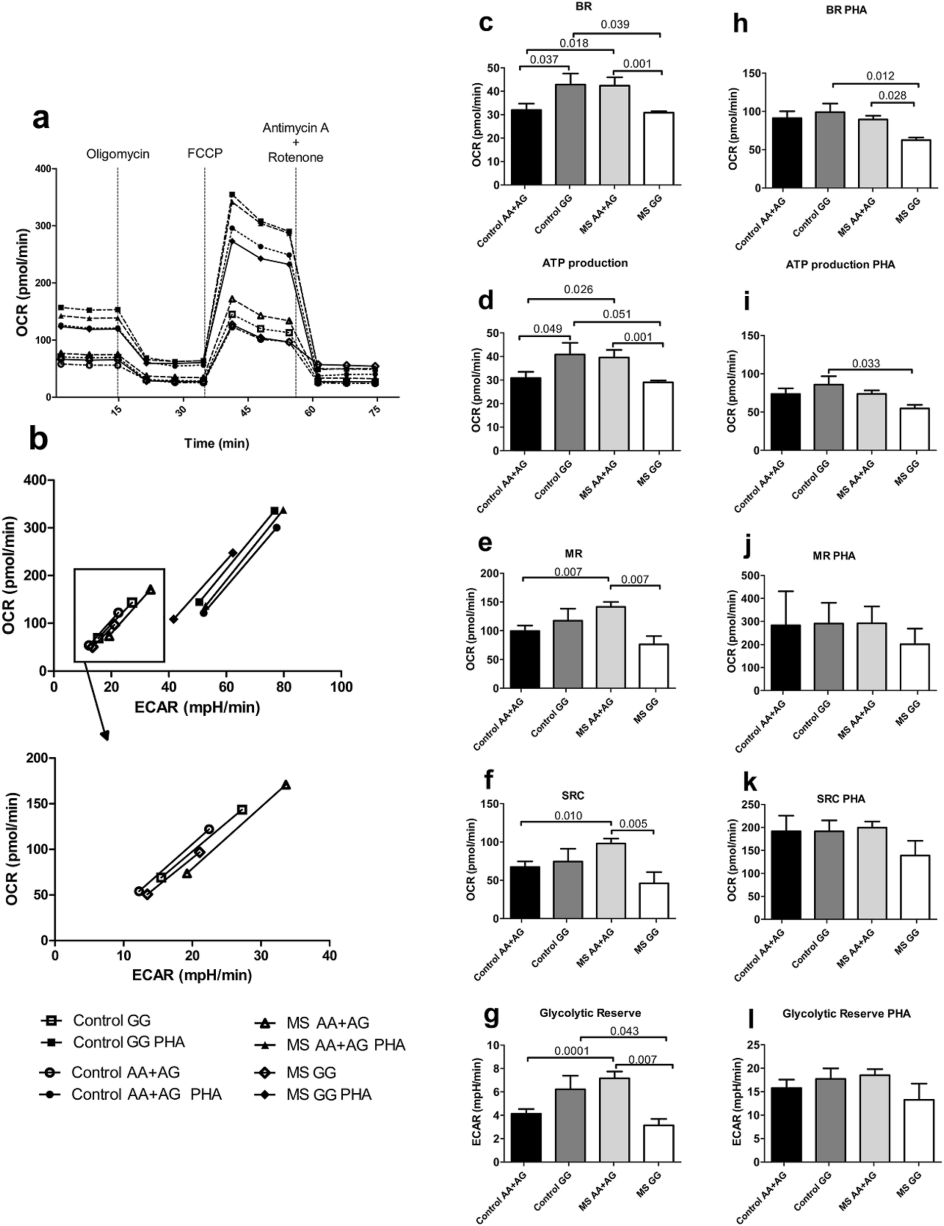
Effect of rs4912622 on lymphocyte metabolism. (**A**) Oxygen Consumption Rate (OCR) profile of PBMCs stimulated or not with PHA from controls or MS patients according to their rs4912622 genotype. (**B**) Energy Phenotype. A zoom out of unstimulated values is shown to improve visibility. (**C-G**) Basal Respiration (BR), ATP Production, Maximal Respiration (MR), Spare respiratory capacity (SRC), and Glycolytic reserve of unstimulated PBMCs. (**H-L**) The same parameters as in (**C-G**) are represented for PBMCs stimulated with PHA. Means ± standard deviations are shown (controls AA + AG: n = 12; controls GG: n = 7; MS AA + AG: n = 21; MS GG: n = 4).

Upon activation, lymphocytes undergo metabolic conversion and thus, we studied the metabolic status through Seahorse technology in both basal and stimulated lymphocytes stratified by the *NDFIP1* variant genotypes (Fig. 4). Oxygen consumption rate (OCR) curves and energy phenotype of unstimulated and PHA-activated samples are presented in Fig. 4A,B. Prior to PHA stimulation, PBMCs from minor-allele homozygous controls showed significantly higher metabolic activity compared to carriers of the major allele, as revealed in both basal mitochondrial respiration and oxidative ATP production (Fig. 4C,D). Glycolytic activity, measured via extracellular acidification rate, also evidenced this trend (Fig. 4G). Lymphocytes from minor-allele homozygous MS patients consistently showed lower metabolic activity than their healthy control counterparts, and significantly lower activity than patients carriers of the major allele. These differences were maintained in most respiratory parameters, as well as in the glycolytic reserve prior to PHA stimulation (Fig. 4C-G). After PHA stimulation, controls and patients seemed to reach a threshold, but minor allele homozygous patients were repeatedly found lagging behind (Fig. 4B,H-L).

## Discussion

The characterization of biological mechanisms underlying genetic risk variants associated with complex traits has proven to be an enormous challenge. The vast majority of GWAS polymorphisms are present in noncoding DNA sequences and are inherited as dense haploblocks. The integrative analyses of eQTL and GWAS data with additional layers of information, such as the expression of transcriptional regulators and other relevant epigenetic cues assist in detecting potentially causal genes. However, the epigenetic information usually pertains to healthy individuals, and it has been found altered in complex diseases, therefore warranting further follow-up studies as ours.

The rs4912622 variant is located within the first intron of *NDFIP1* and presents perfect linkage disequilibrium (D’ = 1 and r^2^ = 1) with the nearby MS-risk SNP, rs1036207. A recent GWAS on MS^2^ highlighted another SNP that displays r^2^ = 0.90 with both rs1036207 and rs4912622, and lies in the 3’ region of *NDFIP1*, rs249677. However, “in silico” data of the latter is not so informative, suggesting that rs249677 is a good MS-risk genetic marker, but it is not the most likely etiologic polymorphism for MS. In contrast, the region surrounding rs4912622 is rich in H3K4me1 and H3K27ac histone marks, according to The Roadmap Epigenomics Project^17^ data. H3K4me1 is characteristic of enhancers^19^ and it might potentiate chromatin accessibility, acting as a platform for chromatin modifiers^20^. The overlapping presence of H3K27ac suggests that the region surrounding rs4912622 could act as an active enhancer^21^ and seems to be important for *NDFIP1* transcriptional regulation. Moreover, data provided by the Genotype-Tissue Expression Project (GTEx) show that, as usually happens, rs4912622 displays different effects depending on the tissue: whereas the GG genotype is associated with a decreased *NDFIP1* mRNA expression in EBV-transformed lymphocytes, an opposite effect is described in whole blood samples, consistent with the data reported by Ricaño et al.^6^ and our own findings in controls (Fig. 2B). It is well known that susceptibility variants exhibit different effects in different tissues, and so it has been suggested that the outcome observed in healthy people may be modified in a pathological context, as distinctive responses of eQTLs depending on the stimulus have been already demonstrated^22^.

In the present study, we observed a significant increase in *NDFIP1* mRNA in MS patients, regardless of treatment, compared to controls. At a protein level, a specular pattern with respect to the mRNA content was evidenced, probably indicative of compensatory mechanisms. A significant decrease in the protein levels was detected in minor-allele homozygous controls, and lower NDFIP1 content was evidenced for MS carriers of the major allele too. Although it has been generally assumed that a direct correlation exists between mRNA and protein levels, only around 40% of the variance in protein expression can be explained by changes at the transcript level^23^, and different protein degradation rates, and post-transcriptional or translational modifications are frequently reported^24^.

As mentioned, NDFIP functions as regulatory proteins of Nedd4-family E3 ligases^11,12^. Herpes simplex virus (HSV) is a common neurotropic virus that is capable of long latencies, can cause focal demyelination, and it has been described more frequently in MS cases than controls^25^. The HSV-2 protein UL56 is also a regulatory protein of Nedd4 E3 ligases, specifically involved in protein stability, and promotes proteasome-mediated degradation by increasing ubiquitination of Nedd4^26^. Moreover, both proteins UL56 and NDFIP1 revealed similar subcellular localization. Therefore, it is tempting to speculate that in MS patients with a reported higher HSV positivity than in controls, a competitive modulatory role of UL56 vs. NDFIP1 could rise.

Several studies have recently highlighted the importance of CD69 expression in immune-mediated diseases and inflammation: blocking or lower expression of CD69 in T cells produces an exacerbation of autoimmune conditions^27^, while CD69^+^ T regulatory cells confer a high immunosuppressive effect in animal models of ulcerative colitis^28^. Concerning the immune activation measured as percentage of CD69^+^ cells after PHA stimulation, we detected significantly higher levels in minor-allele homozygous controls compared to carriers of the major allele for the different cell types studied (Fig. 3). Concordantly, *Ndfip1*(-/-) T cells in mice were activated, and they proliferated and adopted a Th2 phenotype more readily than did their *Ndfip1* (+/+) counterparts^12^. In contrast, this allele-specific activation is lost in MS patients.

The function of *NDFIP1* has been mainly studied in mouse models, and roles in the control of Th17 cell proliferation and Treg cell balance have been reported^11,13,29^. NDFIP1 participates in CD4 and CD8 T-cell regulation in response to high dose antigens. However, its contribution to other processes in the context of MS pathogenesis, such as infection or tolerance to low-dose antigens, have not been so comprehensively described^13^, and no studies in MS patients have been performed to date. As already mentioned, the importance of metabolic programs in cellular activation and cell fate decisions is well recognized^15^. Tregs preferentially rely on lipid oxidation in contrast with effector T cells, which primarily utilize glycolysis as a means of energy generation. Our metabolic analysis consistenly displayed higher levels of basal respiration, maximal respiration, ATP production and a higher glycolytic reserve in unstimulated minor-allele homozygous controls compared to carriers of the major allele (Fig. 4c-g). This results paralelled CD69 expression after activation with PHA (Fig. 3), indicating a stronger potential for immune activation in rs4912622*GG controls. In MS patients, the higher-activity status was present in major allele carriers, but minor-allele homozygotes never reached those levels, indicating a perturbed modulatory role associated to rs4912622 in disease (Fig. 4c-g). The lower activity in MS minor-allele homozygotes compared to controls was maintained in basal respiration and ATP production even after stimulation with PHA (Fig. 4h-i), reenforcing the evidence that the MS GG patients have a defective mitochondrial activation. Interestingly, both the glycolytic and mitochondrial profiles of the allele-stratified cases and controls (Fig. 4g) are specular images of the NDFIP1 protein levels (Fig. 2c), most probably reflecting its key metabolic regulatory role. Even after PHA stimulation, when a threshold in the different mitochondrial parameters seems to be reached by all controls and MS patients, minor-allele homozygous patients are consistently left behind, unable to activate adequately (Fig. 4h-k). Treg cell metabolism has been described dynamically regulated by TLR signals and Foxp3 to balance anabolic metabolism and proliferation with suppressive capacity^30^. Metabolic changes in acute infection or inflammation provide signals that limit the suppressive capacity of Treg cells. As the inflammatory signals decrease, the metabolic balance favours mitochondrial oxidative pathways. This shift would decrease proliferation but would increase the suppressive capacity and promote inflammatory resolution. The suppressive activity of Treg cells in vivo improves protection from experimental autoimmune encephalomyelitis, the MS animal model^31^. However, Treg cells often accumulate but fail to suppress inflammation in chronic autoimmune diseases, as shown in systemic lupus erythematosus or rheumatoid arthritis^32,33^. It has been described that Ndfip1 maintains lineage identity in Treg cells and prevents them from aberrant acquisition of effector T-cell function^29^. While generally stable, Treg cells can have a high degree of instability in inflammatory settings, characterized by the loss of suppressive function, loss of Foxp3 protein or gain of pro-inflammatory IL-4 cytokine production. The increased T-cell proliferation has been found associated with high glycolytic activity and with metabolic programmes that can fuel an effector function in Treg cells and contribute to lineage instability. The reported IL-4 production might have a critical impact in MS patients with an already altered Th1/Th2 balance and the role of NDFIP1 as a molecular sentinel against MS may well depend on variants in the gene, as shown in the present work. Understanding the metabolic signalling might define new regulatory mechanisms and potential targets to modulate immunity. Our data suggest NDFIP1 to be a key metabolic modulator in MS, with an allele-specific metabolic profile completely distinct to the one observed in healthy controls.

In conclusion, our study points at a differential role of the *NDFIP1* risk polymorphism in healthy controls and MS patients. This is an interesting finding provided that many studies on eQTLs are performed in healthy subjects, but as our study and those from others sufficiently prove, polymorphisms may have different effects in different conditions. Targeting metabolic pathways might provide an additional way to control T cell fate in this autoimmune condition and contribute to enhance the effectiveness of actual therapies.

## Materials and methods

### Study population

The study included a total of 87 MS patients (52.9% females) and 84 healthy controls (58.3% females), all Caucasian individuals with mean ages of 43.7 ± 8.1 and 41.1 ± 13.5 years, respectively. Participants were recruited from hospitals of Madrid metropolitan area. All patients were diagnosed with relapsing remitting multiple sclerosis (RRMS) established according to McDonald's criteria^34^. Patients treated with interferon-β or glatiramer acetate were selected due to the difficulty of recruiting enough MS patients without treatment. Two treatments were included in the study in order to confirm or discard any influence of a specific treatment in the biological features studied, and in particular interferon-β and glatiramer acetate were selected for being the ones with longer experience, lower side effects, and general good tolerance among patients. None of the control subjects reported first or second degree relatives with any immune-mediated disease.

Considering that this study deals with immune activation and metabolic activity of PBMCs, the presence of active viral infections that could alter the results was checked. The highly prevalent HHV-6 infection, associated with MS, was evaluated by measuring anti-HHV-6 IgM antibodies (ELISA-VIDATEST, Vidia), that indicate the presence of active replication. All patients but two were negative. The two positive patients showed low titers (below 16 units, positive over 11) and performed just as their negative counterparts in the molecular and statistical analyses. Acute seasonal infections and chronic severe infections such as human immunodeficiency virus or hepatitis C virus were previously discarded by the consulting neurologist. Controls were blood donors who passed all the health enquiries required to donate.

All subjects were recruited after written informed consent. The study was conducted according to the guidelines of the Declaration of Helsinki, and approved by the Ethics Committee of Hospital Clinico San Carlos.

PBMCs (peripheral blood mononuclear cells) separated with Lymphoprep were cryopreserved in liquid nitrogen until further analysis. Genomic DNA was extracted from the granulocyte phase following a salting-out procedure, quantified and preserved at -20ºC.

### Genotyping

Genotyping was carried out with TaqMan technology, following manufacturer indications (Taqman assay for rs4912622; C__31664936_10, Life Technologies). Samples were analysed on a 7900HT Fast Real-Time PCR System (Applied Biosystems). Both controls and MS patients fulfilled the frequencies expected by the Hardy–Weinberg equilibrium.

### Gene expression

Total RNA was isolated from PBMCs with Trizol reagent following the manufacturer’s protocol (Invitrogen, Carlsbad, USA), quantified in a NanoDrop ND-1000 spectrophotometer (NanoDrop Products; Wilmington, DE, USA), and reverse transcribed to cDNA using the High capacity RNA-to-cDNA kit (Applied Biosystems). *NDFIP1* expression was analysed with Taqman probes, using *GUSB* as a housekeeping gene (Applied Biosystems, *NDFIP1*: Hs00242160; *GUSB*: Hs99999908) in a 7900HT Fast Real-Time PCR System with DataAssist v3.01 software (Applied Biosystems).

### Western blot

Twenty controls and 42 patients were included. PBMCs at a concentration of 5 × 10^5^ cells in 10 μl of SDS-PAGE sample buffer (100 mm Tris/HCl, pH 6.8, 3% SDS, 1 mM EDTA, 2% 2-β-mercaptoethanol, 5% glycerol) were analysed by western blot. PVDF membranes were incubated with anti-tubuline (600-401-880, Tebubio) and anti-NDFIP1 (HPA009682, Sigma Aldrich) primary antibodies, followed by anti-rabbit HRP-conjugated secondary antibody (A6154, SIGMA), and visualized with an ECL substrate detection system (Biorad). Blot quantification was performed using Fiji software^35^.

### Flow cytometry

Sixteen controls and 24 patients were included. PBMCs (3 × 10^5^) were stimulated with 10 μg/ml of PHA (L1668, Sigma-Aldrich, Bremen, Germany) and the corresponding unstimulated controls were included for each assay. Activation was assessed 17 h later and cells were stained with anti-CD69-FITC (clone FN50), anti-CD3-PE (clone HIT3a), anti-CD20-APC (clone 2H7), and 7-AAD to exclude non-viable cells (Biolegend, San Diego, CA). Cells were then analysed in a Gallios flow cytometer (Beckman Coulter, Brea, CA) and data was parsed with Kaluza 2.1 software (Beckman Coulter, Brea, CA).

### Cell metabolism

Nineteen controls and 25 patients were included. Oxygen consumption rate (OCR) and extracellular acidification rate (ECAR) of 2 × 10^5^ PBMCs were analysed in a Seahorse Xfp extracellular flux analyser (Agilent). Cells were studied in a basal state (without stimulus) and after stimulation for 24 h with 5 μg/ml of PHA (Sigma). Subsequently, they were adhered onto wells of poly-D-lysine coated XFp plates for 45 min in XF DMEM media supplemented with glucose (10 mM) and pyruvate (2 mM). Each condition was analysed in triplicate with the Cell Mito Stress Test Kit, according to manufacturer’s instructions. Briefly, OCR and ECAR were measured in basal conditions and after oligomycin (1.5 μM), FCCP (0.5 μM), and a cocktail of rotenone and antimycin A (both at 0.5 μM) injections.

### Statistical analysis

Normality was assessed with Kolmogorov–Smirnov test. Normal variables were analysed with Student-t and Anova tests, while variables that did not adjust to normal distribution were analysed with non-parametric Mann–Whitney U test or Kruskal–Wallis test. Statistical analyses were performed with SPSS v15.0.1 (Chicago, Illinois, USA) and outliers were detected with Grubbs' test in GraphPad online tool (https://www.graphpad.com/quickcalcs/Grubbs1.cfm).

## Supplementary Information

Supplementary Information 1.

Supplementary Information 2.

## Data Availability

Data available on request from the authors.

## References

[1] Tillery EE, Clements JN, Howard Z (2017). What's new in multiple sclerosis?. Mental Health Clin..

[2] IMSGC (2019). Multiple sclerosis genomic map implicates peripheral immune cells and microglia in susceptibility. Science.

[3] Olsson T, Barcellos LF, Alfredsson L (2017). Interactions between genetic, lifestyle and environmental risk factors for multiple sclerosis. Nat. Rev. Neurol..

[4] Beecham AH (2013). Analysis of immune-related loci identifies 48 new susceptibility variants for multiple sclerosis. Nat. Genet..

[5] Franke A (2010). Genome-wide meta-analysis increases to 71 the number of confirmed Crohn's disease susceptibility loci. Nat. Genet..

[6] Ricaño-Ponce I (2016). Refined mapping of autoimmune disease associated genetic variants with gene expression suggests an important role for non-coding RNAs. J. Autoimmun..

[7] George AJ, Hoffiz YC, Charles AJ, Zhu Y, Mabb AM (2018). A comprehensive atlas of E3 ubiquitin ligase mutations in neurological disorders. Front. Genet..

[8] Hammond VE (2014). Ndfip1 is required for the development of pyramidal neuron dendrites and spines in the neocortex. Cerebral Cortex (New York, N.Y. : 1991).

[9] Tian J, Zheng W, Li XL, Cui YH, Wang ZY (2018). Lower expression of Ndfip1 is associated with Alzheimer disease pathogenesis through decreasing DMT1 degradation and increasing iron influx. Front. Aging Neurosci..

[10] Sang Q (2006). Nedd4-WW domain-binding protein 5 (Ndfip1) is associated with neuronal survival after acute cortical brain injury. J. Neurosci..

[11] Altin JA (2014). Ndfip1 mediates peripheral tolerance to self and exogenous antigen by inducing cell cycle exit in responding CD4+ T cells. Proc. Natl. Acad. Sci. U.S.A..

[12] Oliver PM (2006). Ndfip1 protein promotes the function of itch ubiquitin ligase to prevent T cell activation and T helper 2 cell-mediated inflammation. Immunity.

[13] Wagle MV (2018). The ubiquitin ligase adaptor NDFIP1 selectively enforces a CD8(+) T cell tolerance checkpoint to high-dose antigen. Cell Rep..

[14] Kleinewietfeld M, Hafler DA (2014). Regulatory T cells in autoimmune neuroinflammation. Immunol. Rev..

[15] Michalek RD (2011). Cutting edge: Distinct glycolytic and lipid oxidative metabolic programs are essential for effector and regulatory CD4+ T cell subsets. J. Immunol. (Baltimore, Md.: 1950).

[16] Boyle AP (2012). Annotation of functional variation in personal genomes using RegulomeDB. Genome Res..

[17] The Roadmap Epigenomics Project. http://www.roadmapepigenomics.org/.

[18] Auton A (2015). A global reference for human genetic variation. Nature.

[19] Rada-Iglesias A (2018). Is H3K4me1 at enhancers correlative or causative?. Nat. Genet..

[20] Local A (2018). Identification of H3K4me1-associated proteins at mammalian enhancers. Nat. Genet..

[21] Creyghton MP (2010). Histone H3K27ac separates active from poised enhancers and predicts developmental state. Proc. Natl. Acad. Sci. U.S.A..

[22] Lee MN (2014). Common genetic variants modulate pathogen-sensing responses in human dendritic cells. Science (New York, N.Y.).

[23] de Sousa Abreu R, Penalva LO, Marcotte EM, Vogel C (2009). Global signatures of protein and mRNA expression levels. Mol. bioSyst..

[24] Van Nostrand EL (2020). A large-scale binding and functional map of human RNA-binding proteins. Nature.

[25] Xu L (2021). Positive association of herpes simplex virus-IgG with multiple sclerosis: A systematic review and meta-analysis. Multiple sclerosis and related disorders.

[26] Ushijima Y (2010). Herpes simplex virus UL56 interacts with and regulates the Nedd4-family ubiquitin ligase Itch. Virol. J..

[27] Gonzalez-Amaro R, Cortes JR, Sanchez-Madrid F, Martin P (2013). Is CD69 an effective brake to control inflammatory diseases?. Trends Mol. Med..

[28] Yu L (2018). CD69 enhances immunosuppressive function of regulatory T-cells and attenuates colitis by prompting IL-10 production. Cell. Death Dis..

[29] Layman AAK (2017). Ndfip1 restricts mTORC1 signalling and glycolysis in regulatory T cells to prevent autoinflammatory disease. Nat. Commun..

[30] Gerriets VA (2016). Foxp3 and Toll-like receptor signaling balance T(reg) cell anabolic metabolism for suppression. Nat. Immunol..

[31] Gerriets VA (2015). Metabolic programming and PDHK1 control CD4+ T cell subsets and inflammation. J. Clin. Investig..

[32] Ohl K, Tenbrock K (2015). Regulatory T cells in systemic lupus erythematosus. Eur. J. Immunol..

[33] Byng-Maddick R, Ehrenstein MR (2015). The impact of biological therapy on regulatory T cells in rheumatoid arthritis. Rheumatology (Oxford).

[34] Polman CH (2011). Diagnostic criteria for multiple sclerosis: 2010 revisions to the McDonald criteria. Ann. Neurol..

[35] Schindelin J (2012). Fiji: An open-source platform for biological-image analysis. Nat. Methods.

