# Supplementary figures and images for: Genetic variation in NDFIP1 modifies the metabolic patterns in immune cells of multiple sclerosis patients
Source: Sci Rep. 2021 Nov 1;11:21371. doi: 10.1038/s41598-021-00528-8 (PMC8560952; doi:10.1038/s41598-021-00528-8)

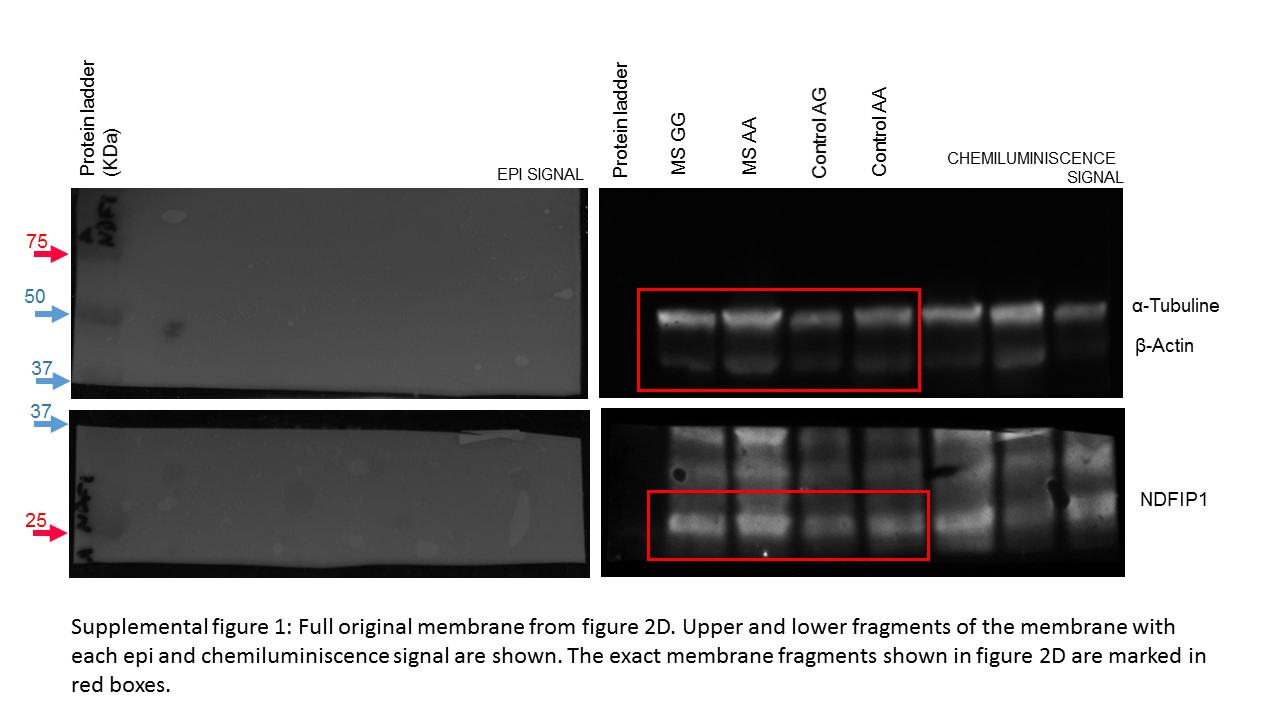

Supplement: Supplementary file 1 — Supplementary Information 1. [file 41598_2021_528_MOESM1_ESM.jpg]
